# Evaluation of the lacrimal and nasolacrimal system in dogs treated at a veterinary ophthalmology service

**DOI:** 10.29374/2527-2179.bjvm001125

**Published:** 2025-05-28

**Authors:** Lucas de Souza Viana, Diego Neves Vianna, Camila Cristina Rio Preto Martins de Sousa, Suzana de Souza Lima, Bruno Alberigi

**Affiliations:** 1 Programa de Residência em Medicina Veterinária – Oftalmologia de Animais de Companhia. Hospital Veterinário (HV), Instituto de Veterinária (IV), Universidade Federal Rural do Rio de Janeiro (UFRRJ). Seropédica, RJ, Brazil.; 2 Autonomus Veterinarian, São Luís, MA, Brazil.; 3 Departamento de Medicina e Cirurgia Veterinária (DMCV), IV, UFRRJ. Seropédica, RJ, Brazil.

**Keywords:** ocular surface, precorneal tear film, tear crystallization, tear production, superfície ocular, filme lacrimal pré-corneano, cristalização da lágrima, produção lacrimal

## Abstract

In routine veterinary medicine, the lacrimal and nasolacrimal systems are associated with several ophthalmopathies. Understanding its physiology and improving specific diagnostic tests will help establish an assertive approach and avoid lacrimal and nasolacrimal disorders that cause damage to the ocular surface. This prospective study was conducted on 43 dogs treated at the Veterinary Ophthalmology Service and approved by the Ethics Committee for the Use of Animals under protocol number 5154141022. The study's objective of this study was to evaluate lacrimal and nasolacrimal system findings in dogs treated byat an ophthalmology service, determine the frequency of lacrimal and nasolacrimal system disorders, and correlate the diagnosed changes with the patient's main complaint. All dogs underwent a complete ophthalmic examination, emphasizing the lacrimal and nasolacrimal tests: the Schirmer tear test, tear film break-up test, tear crystallization test, and Jones test. The findings of these tests correlated with those of the ocular surface and the general condition of the dogs. Statistical analyses were performed using the Shapiro–Wilk test, Pearson's chi-square test, and Student's *t* test. The Student's *t* test revealed that dogs with normal tear crystallization test scores had a significantly higher score (25.5 ± 4.94) than dogs with altered tear crystallization test scores (15.35 ± 6.64) (*t* (40) = 2.121, *p* = 0.004). Our findings suggest that quantitative and qualitative tear tests should be performed together and their interpretation depends on several factors, including ocular and systemic disorders.

## Resumo

Na rotina da medicina veterinária, os sistemas lacrimal e nasolacrimal estão associados a diversas oftalmopatias. Compreender sua fisiologia e aprimorar testes diagnósticos específicos auxiliará no estabelecimento de uma abordagem assertiva e na prevenção de distúrbios lacrimais e nasolacrimais que causam danos à superfície ocular. Este estudo prospectivo foi realizado em 43 cães atendidos no Serviço de Oftalmologia Veterinária, com aprovação do Comitê de Ética no Uso de Animais sob o protocolo número 5154141022. O objetivo do estudo foi avaliar as alterações no sistema lacrimal e nasolacrimal em cães atendidos em um serviço de oftalmologia, determinar a frequência de distúrbios nesses sistemas e correlacionar as alterações diagnosticadas com a queixa principal do paciente. Todos os cães foram submetidos a um exame oftálmico completo, com ênfase nos testes lacrimais e nasolacrimais: teste de Schirmer, teste de ruptura do filme lacrimal, teste de cristalização da lágrima e teste de Jones. Os achados desses testes foram correlacionados com a avaliação da superfície ocular e a condição geral dos cães. As análises estatísticas foram realizadas utilizando o teste de Shapiro–Wilk, o teste do qui-quadrado de Pearson e o teste t de Student. O teste t de Student revelou que cães com cristalização lacrimal normal apresentaram uma pontuação significativamente maior (25,5 ± 4,94) do que cães com cristalização lacrimal alterada (15,35 ± 6,64) (t(40) = 2,121, p = 0,004). Nossos resultados sugerem que os testes quantitativos e qualitativos da lágrima devem ser realizados em conjunto, e sua interpretação depende de vários fatores, incluindo alterações oculares e sistêmicas.

## Introduction

The lacrimal and nasolacrimal systems represents a set of structures responsible for the production and drainage of tears, also called the precorneal tear film (PCTF). Effective tear dynamics and a well-balanced PCTF composition are critical for maintaining homeostasis and physiology of the ocular surface (Sebbag & Mochel, 2020). Composed of three layers, the PCTF maintains an optically uniform corneal surface, removes foreign material and debris from the cornea and conjunctival sac, provides oxygen to the avascular cornea, and supplies antimicrobial substances (Meekins et al., 2021). The failure of this system can be observed through diagnostic tests during ophthalmic examinations.

The Schirmer tear, performed for quantitative analysis of the aqueous portion of the tear, is the most widely used test in clinical routine; it consists of millimeter-scale strips of specific dimensions whose ends are inserted into the lower conjunctival sac of each eye (Bertram et al., 2021). Qualitative methods, such as tear crystallization tests and tear film break-up times, are used to evaluate the lipid and mucosal portions of the PCTF. Tear crystallization is a simple and low-cost test that involves depositing a drop of tears on a microscope slide and waiting for it to dry to characterize the crystallization pattern and consequent quality according to grading scales (Oriá et al., 2018). The tear film break-up time was assessed when the PCTF presents dry spots immediately after the instillation of a drop of fluorescein; the average time in a healthy dog varied between 15 and 20 s. This methods enables the identification of qualitative changes in tears, even if their quantity has not been affected (Giuliano, 2021). After being produced, the PCTF must be drained through the drainage system, which is represented by the nasolacrimal duct. The objective of this study was to evaluate the findings of the lacrimal and nasolacrimal systems in dogs treated by an ophthalmology service, determine the frequency of lacrimal and nasolacrimal system disorders, and investigate the correlation between the diagnosed changes in the lacrimal and nasolacrimal systems and the patient's main complaints.

## Material and methods

A prospective study was conducted in 43 dogs treated at the Veterinary Ophthalmology Service of the Veterinary Hospital of Universidade Federal Rural do Rio de Janeiro. These patients were admitted with complaints of ophthalmic diseases, and with no apparent complaints, they were interested in ophthalmic checkups. All individuals responsible for the animals evaluated in this study signed an informed consent form. The exclusion criteria were dogs presenting with corneal perforations, lacerations, or trauma to the ocular adnexa that prevented the examinations from being performed and uncooperative patients who did not allow the ophthalmic examination and the tests to be performed in full.

The records included the identification of the patient and person responsible for the animal, animal's history, anamnesis questions regarding the lacrimal and nasolacrimal systems, classification of corneal surface disorders, results of tests related to the lacrimal and nasolacrimal systems, case diagnosis, requests for additional tests, and prescribed treatment.

Patients underwent individual complete ophthalmic examinations, with emphasis on the lacrimal and nasolacrimal systems. Each eye was inspected for the corneal surface, and the following conditions were classified according to their intensity: corneal pigmentation, corneal edema, epiphora, conjunctival hyperemia, corneal neovascularization, mucous secretion, and mucopurulent secretion. The lacrimal system was evaluated by using quantitative and qualitative tear tests, whereas the nasolacrimal system was evaluated by using the Jones test.

**Figure 1:**
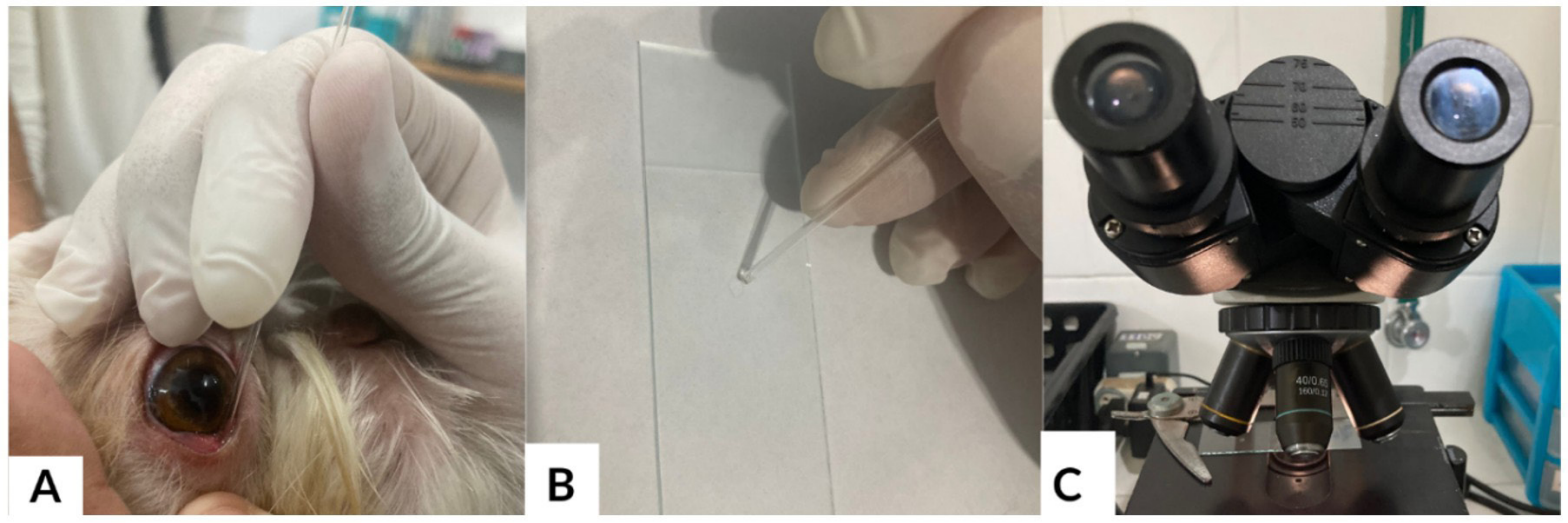
Simplified methodology of the tear crystallization test. Tear collection (A), placement on a slide for drying (B), and subsequent microscopic evaluation (C).

Source: Author's collection.

**Figure 2:**
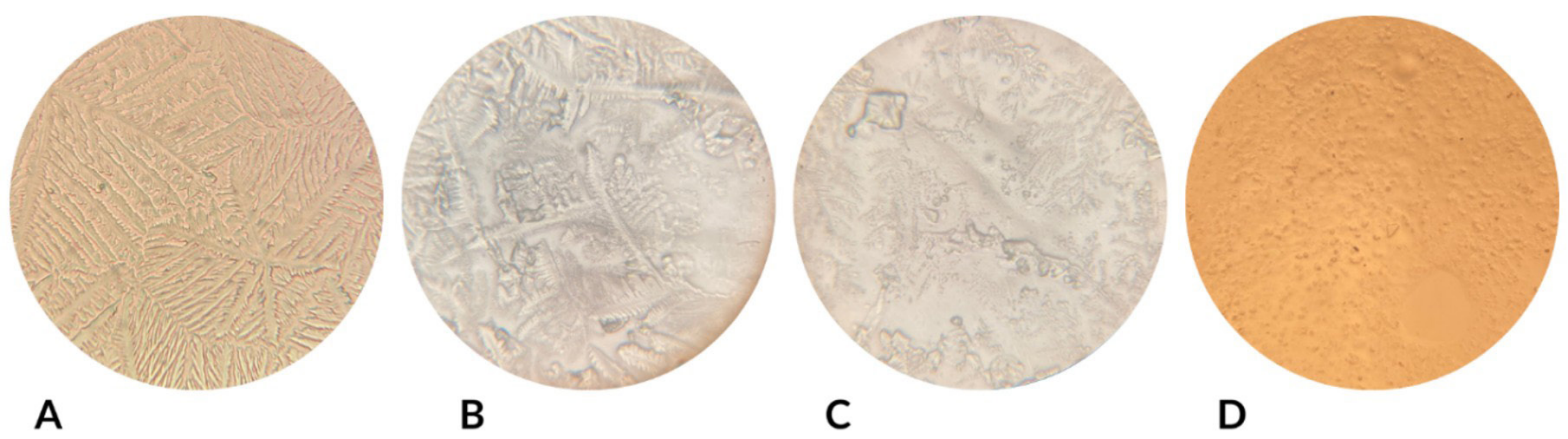
Microscopic images of tear crystallization patterns: types I (A), II (B), III (C), and IV (D).

Source: Author's collection.

The Schirmer Type 1 Tear Test (STT-1) was performed to quantitatively evaluate the precorneal tear film. For each dog, commercially available sterile STT-1 strips were inserted into the inferior conjunctival sac of both eyes (right and left) for 60 s; the wetting of the strips was then quantitatively measured in millimeters. The typical values ranged 15–25 mm/min. Subsequently, a tear crystallization test was performed. A microcapillary was inserted into the inferior conjunctival sac of each eye to collect the tears from each dog's eyes, which were then placed on glass slides (Figure 1). After drying at room temperature, the tear crystallization pattern was qualitatively evaluated by microscopic (Figure 2) examination; the pattern was classified from I to IV according to Masmali et al. (2014).

The qualitative method tear film break-up time test was also performed. Fluorescein eye drops were instilled into both corneas of each dog, and the ability of the corneal surface to retain homogeneous tear coverage and dye breakdown time in pools were subjectively assessed. The PCTF was classified based on the dye breakdown time in pools as follows: 15–20 s, good; 10–14 s, fair; and 1–9 s, bad. The Jones test was used to assess the nasolacrimal system. Fluorescein eye drops were instilled into the eyes of each dog. The nostrils were then inspected within 10 min to assess the patency of the nasolacrimal duct.

In the present study, the normality of the distribution of all variables was assessed using the Shapiro–Wilk test. The frequencies of sex, clinical complaints, age, and breed were analyzed. The frequency of the presence or absence of alterations in the lacrimal system assessments, as well as the absence of an association between the eye (right or left) and presence of alterations in the lacrimal system examinations were assessed using Fisher's exact test. The frequency of such findings and association with the affected eye (right or left) were analyzed using Fisher's exact test.

The chi-square test was used to assess the relationship between the crystallization test results and eye conditions and between the STT-1 results and eye conditions. The Levene test verified the assumption of homogeneity of variance. In cases of equality of variance, the Levene test was used, and when equality of variance was not assumed, the Welsh correction was applied.

To increase the reliability of the results and correct deviations from normality in the sample distribution and differences between the group sizes, bootstrapping procedures were performed with 1000 resamplings and 95% bias-corrected and accelerated confidence intervals. These procedures also allowed a 95% confidence interval for the differences between the means (Haukoos & Lewis, 2005). IBMM SPSS Statistics for Windows, version 29.0.1 (IBM Corp., Armonk, NY, USA) was used for all analyses with a significance level of 95%.

## Results

The Shapiro–Wilk test demonstrated that only age presented a normal distribution (S = 0.972, *gl* = 42, *p = 0.393*).

A total of 43 dogs were evaluated between December 2022 and August 2023, of which 53.5% (23/43) were female and 46.5% (20/43) were male. The mean age of the included dogs was 7.3 ± 4 years; 74.4% (32/43) were under 10 years old, and 25.6% (11/43) were over 10 years old.

**Figure 3:**
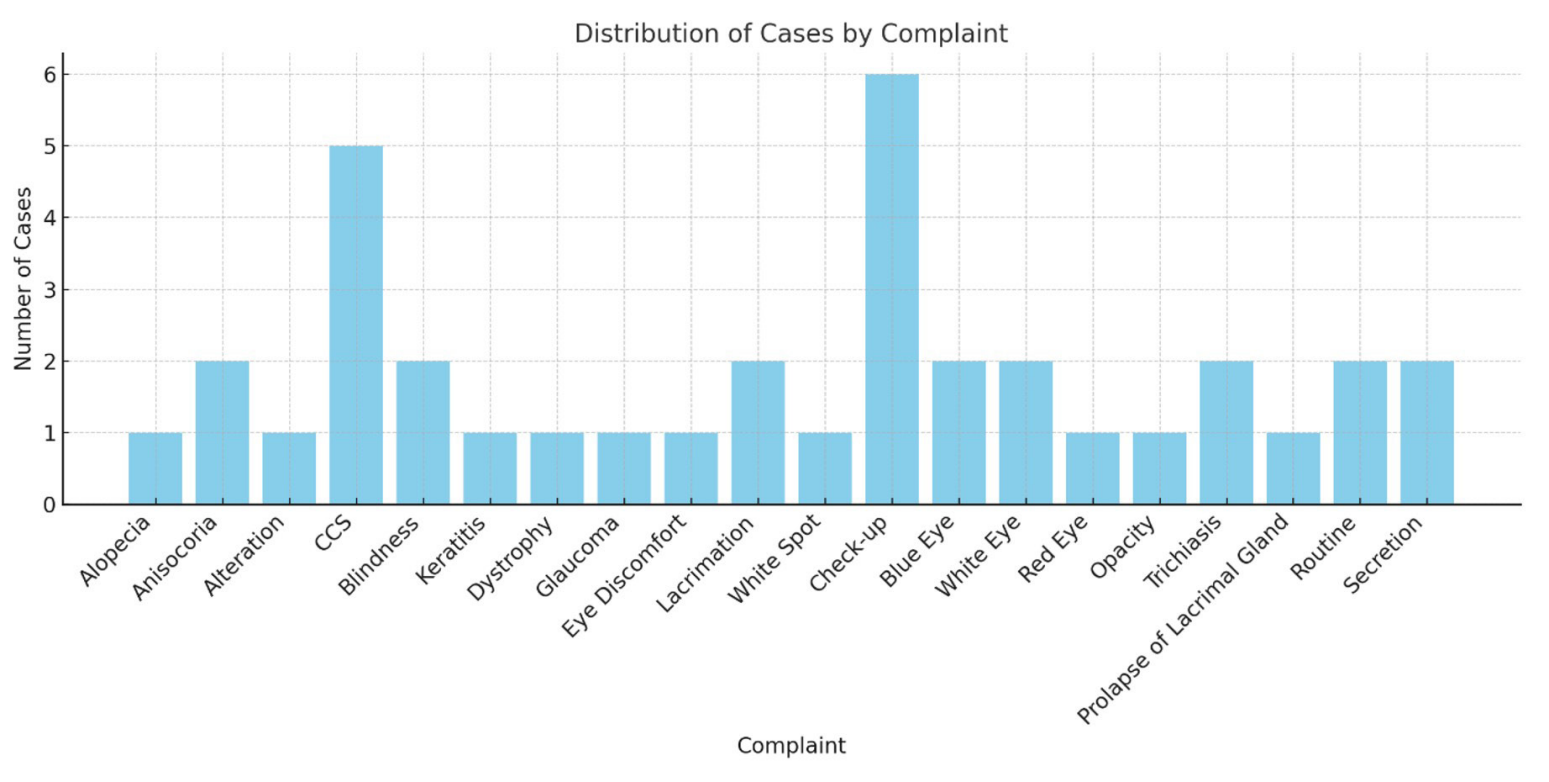
Main complaints for ophthalmic care in dogs.

A total of 43 dogs were evaluated between December 2022 and August, 2023. Dogs were admitted to the ophthalmology department for primary ocular complaints (85%, 37/43) such as corneal opacities (e.g., “whitish eyes” [25%, 11/43]), keratoconjunctivitis sicca (20%, 9/43), and epiphora (4,6%, 2/43) as well as non-ophthalmic systemic conditions (15%, 6/43) requiring ophthalmic evaluation (Figure 3).

The distribution of breeds was as follows: 35% (15/43) mixed breed, 16.3% (7/43) Shih Tzu, 14% (6/43) Poodle, 9.3% (4/43) Pug, and other breeds that each represent <5% of all included dogs.

Therefore, most dogs in the study had a mesocephalic cranial conformation (69.8%), followed by brachycephalic (25.5%) and dolichocephalic (4.7%).

Upon evaluating the lacrimal system, the mean results of the STT-1 for the right and left eyes were the same (15.8 ± 6.8 mm). The frequency of the presence or absence of alterations in the lacrimal system assessments, as well as the absence of an association between the eyes (right and left) and presence or absence of alterations in the lacrimal system evaluation tests (normal or altered), are shown in Table 1. In the right eye, the STT-1 scores were normal in 65.1% (n = 28) and altered in 34.9% (n = 15); the tear film break-up test scores were normal in 37.2% (n = 16) and altered in 62.8% (n = 27); the tear crystallization patterns were normal in 2.3% (n = 1) and altered in 97.7% (n = 42) changed; and the Jones test results were normal in 41.9% (n = 18) and altered in 58,1% (n = 25). In the left eye, the STT-1 scores were normal in 62.8% (n = 27) and altered in 34.9% (n = 15); the tear film break-up test scores were normal in 37.2% (n = 16) and altered in 60.5% (n = 26); the crystallization patterns were normal in 4.7% (n = 2) and altered in 93% (n = 40); and the Jones test results were normal in 39.5% (n = 17) and altered in 60,5% (n = 26). The Pearson's chi-square test results were 0.006 for STT-1, 0.007 for the tear film break-up test, 0.370 for the tear crystallization test, and 0.017 for the Jones test.

**Table 1:** Frequency (regular and altered) of assessments in lacrimal and nasolacrimal tests.

Test	Right eye			Left eye			*X ^2^ (gl)*	*Fisher's exact*
	Normal (n)		Altered (n)	Normal (n)		Altered (n)		
Schirmer tear test	65.1% (28)		34.9% (15)	62.8% (27)		34.9% (15)	0.006 (1)	1.000
								
Break up test	37.2% (16)		62.8% (27)	37.2% (16)		60.5% (26)	0.007 (1)	1.000
								
Crystallization	2.3% (1)		97.7% (42)	4.7% (2)		93% (40)	0.370(1)	0.616
								
Jones test	41.9% (18)		58.1% (25)	39.5% (17)		60.5% (26)	0.017 (1)	1.000

Note: x ^2 =^ Pearson's chi-square; df = degrees of freedom.

Examination of the ocular surface revealed the presence of ocular secretions, epiphora, conjunctival hyperemia, corneal vascularization, and corneal pigmentation. Table 2 shows the frequency of these findings and their association with the affected eye (right or left), with an emphasis on eye discharge (55.8% in the right and left eyes), conjunctival hyperemia (32.6% in the right and left eyes), and corneal vascularization (27.9% in the right eye and 32.6% in the left eye).

**Table 2:** Presence of ophthalmic clinical signs in the dogs.

Changes	Right eye				Left eye			*X ^2^ (gl)*	*Fisher's exact*
	Present (n)		Absent (n)		Present (n)		Absent (n)		
Eye discharge	55.8% (24)		44.2% (19)		55.8% (24)		44.2% (19)	0.653(2)	0.853
									
Epiphora	23.3% (10)		76.7% (33)		23.3% (10)		76.7% (33)	0.754(2)	0.564
									
Conjunctival hyperemia	32.6% (14)		67.4% (29)		32.6% (14)		67.4% (29)	0.781(2)	0.494
									
Corneal vascularization	27.9% (12)		72.1% (31)		32.6% (14)		67.4% (29)	0.755(4)	1.895
									
Corneal Pigmentation	30.2% (13)		69.8% (30)		29.5% (17)		60.5% (26)	0.467(2)	1.843
									
Corneal edema	27.9% (12)		73.8% (31)		25.6% (11)		73.8% (31)	0.818(2)	0.401

The Pearson's chi-square test showed no significant difference between the crystallization tests results and eye conditions (both eyes) or between the STT-1 results and eye conditions (both eyes). Among the clinical signs, the relationship between corneal pigmentation and eye conditions was significant for the right eye (Pearson's chi-square 64.978 and *p* = 0.006) but not for the left eye (Pearson's chi-square 46.229 and *p* = 0.

The Student's *t* test for independent samples was performed to investigate the extent to which the levels of tear production in the right and left eyes, as assessed by quantitative STT-1, differed between males and females, between animals older or younger than 10 years of age, between animals with normal or altered crystallization pattern, and between animals with or without systemic diseases. The results demonstrated that dogs with normal crystallization patterns had a significantly higher score (25.5 ± 4.94) than dogs with altered crystallization patterns (15.35 ± 6.64) (*t* (40) = 2.121, *p* = 0.004), demonstrating a high effect size (Hedge's G = 1.8*).* Significantly higher scores were also observed among dogs without systemic disease (17.40 ± 6.30) than among dogs with systemic disease (11.18 ± 6.25) (*t* (41) = -2.828, *p* = 0.007), with a high effect size (Hedge's G = 0.9) (Table 3).

**Table 3:** Results of the test for difference in the levels of tear production in the right and left eyes, assessed by quantitative TLS, were different between males and females, between animals older or younger than 10 years of age, between animals with normal or altered crystallization, and between animals with or without systemic diseases.

		Scores	Test statistics *(Bootstrapping sample)*					
		*M±SD*	*t*	*Gl*	*p* value	Mean Difference	Mean Difference CI (95%)	
							Limit lower	Upper limit
Tear production	Female	17.5 *±5.4*	1.764	41	0.085	3.58	-0.51	7.67
	Male	13.9 *±7.8*						
	Age >10 years	17.5 *±8.7*	0.978	41	0.334	2.33	-2.47	7.13
	Age < 10 years	15.2 *±6.1*						
	Normal STT-1 results	22 *±0*	0.919	41	0.364	6.33	-7.58	20.25
	Altered STT-1 results	*15.6±6.8*						
	Normal crystallization	*25.5±4.94*	2.121	40	0.040	10.15	0.47	19.82
	Altered crystallization	*15.35±6.64*						
	Systemic disease	*11.18±6.25*	-2.828	41	0.007	-6.22	-10.66	-1.78
	No systemic disease	*17.40±6.30*						

STT-1, Schirmer’s tear test; M: Mean, SD: Standard Deviation; Df: Degrees of freedom; CI: Confidence interval.

## Discussion

The lack of correlation (*p* > 0.05) between age and the lacrimal and nasolacrimal tests (STT-1, tear film break-up test, tear crystallization test, and Jones test) observed in this study is similar to the results of a study by Faghihi and Rajaei (2022), who did not observe a correlation between age and the results of STT-1 and tear film break-up test in either eye (*p* [odds ratio] > 0.05). However, previous studies included heterogeneous populations of different breeds: Faghihi and Rajaei (2022) predominantly analyzed dolichocephalic breeds (e.g., greyhounds), whereas Sebbag et al. (2022) focused on Shih Tzus, a brachycephalic breed with an anatomical predisposition to tear disorders. The differing study populations may explain the variations in the findings, reinforcing the need to consider breed characteristics when interpreting the results. Notably, Sebbag et al. (2022), who focused on the Shih Tzu breed, showed a negative correlation between age and the tear film break-up test results (*r* = −0.31, *p* = 0.027). Further research is necessary to elucidate the potential influence of age on lacrimal system function in dogs.

Upon applying the inclusion and exclusion criteria, we observed 8 breeds among 43 animals included in the study, with a higher prevalence of mixed-breed dogs (35%). This indicated a the typical population of dogs treated at a veterinary hospital—often of mixed or smaller breeds, common in urban environments. Studying these dogs in veterinary care is beneficial, disproving the general belief that mixed-breed dogs are more resistant to diseases than defined breeds and, therefore, do not require veterinary care (Penaforte et al., 2024). The other most prevalent breeds in the present study are known to be predisposed to ophthalmic diseases. The Shih Tzu breed stands out owing to its a high prevalence of ocular surface diseases, often related to lacrimal deficiency and anatomical abnormalities of the ocular adnexa (Sebbag et al., 2022).

The predominance of mesocephalic dogs reflected the general canine population in urban settings, including asymptomatic individuals, which may introduce a selection bias. However, brachycephalic breeds demonstrated a higher predisposition to lacrimal disorders, consistent with their anatomical vulnerabilities such as anomalous canaliculi, tortuous nasolacrimal ducts (Sahr et al., 2021), and exophthalmia-related corneal exposure, which exacerbate epiphora and dry eye risk (Sebbag et al., 2022). Despite efficient drainage in normocephalic dogs (Sahr et al., 2021), the high prevalence of altered tear test results across all groups suggests that lacrimal dysfunction may occur secondary to systemic conditions or other ocular pathologies, even without a direct correlation with surface changes. These findings highlight the need for comprehensive lacrimal assessments in all dogs, regardless of cranial conformation, to address multifactorial etiologies.

The main reasons for ophthalmic care in dogs in the study were check-up consultations in the absence of ocular complaints, followed by whitish eyes and complaints compatible with keratoconjunctivitis sicca. Notably, preventive veterinary medicine in real-word settings should be performed excellently and based on scientific evidence. To this end, theories, models, and structures used in human medicine are good alternatives for the better absorption of preventive practices based on scientific evidence in veterinary medicine (Reyneke et al., 2023).

Whitish eyes are one of the many possible causes of ocular opacity and may be present on the corneal surface or in intraocular structures. When treating dogs with such complaints from their owners, investigations of corneal opacities and cataracts, which are prominent in the clinical routine, should be performed (Malik & Pandey, 2021). Keratoconjunctivitis sicca was the motivation for the main tests performed in this study owing to its direct association with the quantity and quality of the lacrimal system. The clinical signs of this disease include uncomfortable eyes that are difficult to manage, mucopurulent secretion, and a general dry appearance of the cornea (Woodham-Davies, 2020).

Our findings indicated that alterations in the lacrimal system (e.g., reduced STT-1 and abnormal crystallization) did not correlate directly with ocular surface abnormalities (*p* > 0.05), corroborating previous findings that highlighted the multifactorial nature of ocular dysfunction in dogs (Sebbag et al., 2022; Woodham-Davies, 2020). For example, in Shih Tzus, even with adequate tear production, exophthalmos and chronic corneal exposure can trigger independent inflammatory conditions (Sahr et al., 2021). This supports the notion that assessing the lacrimal system alone is insufficient to predict changes in the ocular surface, and anatomical and systemic factors must be considered.

Most ophthalmic clinical signs did not significantly differ between the eyes, except for corneal pigmentation, which was significantly associated with ocular disorders only in the right eye. This finding may indicate that corneal pigmentation, which is characterized by the accumulation of melanin, melanocytes, or melanophages within and under the corneal epithelium (Labelle & Labelle, 2023), has a unilateral tendency under certain conditions. However, the reason for this unilateral difference requires further investigation.

Student's *t* tests indicated that normal tear crystallization and the absence of systemic diseases were associated with greater tear production. We found that dogs with systemic diseases had a lower tear production index, suggesting that systemic conditions and abnormalities in tear quality (such as crystallization) can significantly affect tear function in dogs. Dogs with sufficient tear quality and quantity are likely to have a healthy ocular surface, because the PCTF is described as an overlap of beds with lipid, aqueous, and mucous components (Galera & Cremonini, 2023). Furthermore, most dogs showed reduced tear film break-up time, indicating an unstable tear film and suggesting that this examination should be performed routinely.

Most dogs in the study presented with altered crystallization, suggesting that crystallization may be the first test to indicate lacrimal disease in patients and that its use should be disseminated. However, this result may represent a limitation of our study, as tear drying was performed at an ambient temperature. Oriá et al. (2018) described drying tears at room temperature and monitored temperature and humidity, whether collected using a microcapillary (as in our study), micropipette, or centrifuged STT-1 strips. Tang et al. (2020) collected tears from mice and dried them in an oven at controlled temperature and humidity. Future studies should compare different methods of tear drying for crystallization in dogs, as while we believe that drying at room temperature is the easiest way to reproduce in a clinical routine, it can also be affected by the climate.

Regarding evaluation of the nasolacrimal system, represented by the Jones test, higher percentages of altered results (58.1% in the right eye and 60.5% in the left eye) were observed in this study. The observed failure lacrimal drainage may be attributed to anatomical factors, as brachycephalic dogs such as Shih Tzus, Pugs, and French Bulldogs were free. The nasolacrimal duct of dogs is long; its anatomy depends on the cephalic conformation, which varies between dolichocephalic and brachycephalic breeds, and brachycephalic breeds are the most susceptible to nasolacrimal disorders (Ali et al., 2019). Brachycephalic dogs may present with malformation and a U- or V-shaped anomalous appearance of the nasolacrimal apparatus, with tortuosity of the nasolacrimal duct and an inverse direction of the lacrimal canaliculi (Sahr et al., 2021). Poodles, another prevalent breed in this study, commonly present with nasolacrimal duct atresia, causing partial or total failure of tear drainage, although this diagnosis was not investigated during the study (Pickett, 2019).

In the present study, the high frequency of altered lacrimal and nasolacrimal system test results indicate that dogs in this population have a high prevalence of lacrimal and nasolacrimal system dysfunction. Such dysfunctions may contribute to the development of ocular surface changes (Ali et al., 2019; Sebbag et al., 2022) or be a consequence of other ocular abnormalities or systemic diseases (Woodham-Davies, 2020).

The present results reinforce the fact that lacrimal system disorders are common in dogs (Marchini et al., 2024) and highlight the importance of thoroughly evaluating the lacrimal system in dogs with ocular surface abnormalities. The statistically significant association between altered tear crystallization and decreased tear production suggests that the tear crystallization test may be a useful for assessing lacrimal system function in dogs and should be performed in routine clinical practice. Additionally, the finding that dogs with systemic diseases have lower tear production than healthy dogs highlights the potential impact of systemic conditions on lacrimal system function, as reported in other studies (Naranjo et al., 2005; Wegg, 2020; Woodham-Davies, 2020).

The high prevalence of dogs without ocular complaints (15% of the sample) that exhibited altered tear test results suggests that subclinical dysfunction may precede visible manifestations, consistent with reports in humans (Masmali et al., 2014) and canines (Oriá et al., 2018). For instance, Giuliano (2021) observed a progressive reduction in tear film break-up time in asymptomatic elderly dogs, indicating that qualitative changes in tears may be an early risk marker of ocular surface diseases. Our results reinforce the need to include tear tests in routine checkups, even in the absence of clinical signs. While many dogs in this study had abnormal lacrimal system test results, ocular surface changes such as conjunctival hyperemia, corneal vascularization, and corneal pigmentation were not directly related to the lacrimal system test results. Therefore, lacrimal system dysfunction may not necessarily lead to ocular surface abnormalities, or that other factors, such as underlying systemic or ocular diseases, may play a more significant role in developing these changes.

The present study has some limitations, such as the relatively small sample size and lack of information on the duration and severity of lacrimal system dysfunction; nevertheless, it highlighted the importance of lacrimal and nasolacrimal assessments in dogs with or without ocular complaints.

## Conclusions

In this study, we evaluated the lacrimal and nasolacrimal systems in dogs, highlighting their combined value in diagnosing canine ophthalmic diseases. The results showed a high prevalence of abnormalities in both systems, such as tear crystallization, decreased tear film break-up time, and possible dysfunction of tear drainage through the nasolacrimal pathway.

A high prevalence of alterations in the lacrimal and nasolacrimal systems was identified in dogs treated by a veterinary ophthalmology service, highlighting the usefulness of the tear crystallization test as a complementary diagnostic tool. However, the absence of a significant association between these alterations and ocular surface abnormalities suggests that systemic factors and anatomical predispositions such as those observed in brachycephalic breeds may play important roles in the pathophysiology of these conditions. Notably, causal inferences require caution due to the moderate sample size and heterogeneity of the population studied. Prospective studies with larger cohorts and controls stratified by breeds are needed to validate these findings and explore the underlying mechanisms.

The tear crystallization test is a potential diagnostic tool that strongly correlates with tear production and should be performed frequently. Additionally, standardizing methodologies for tear crystallization testing and further exploring the effects of environmental factors and unilateral variability in conditions such as corneal pigmentation are necessary. By incorporating these insights into veterinary practice, clinicians can improve the diagnostic precision and better manage disorders involving the lacrimal and nasolacrimal systems.
